# Association of recurrent laryngeal nerve lymph node retrieval with survival in early-stage resectable esophageal squamous cell carcinoma: a retrospective cohort study

**DOI:** 10.7717/peerj.21293

**Published:** 2026-06-04

**Authors:** Qiuchen Ge, Bianyin Sun, Yuting Sang, Tao Lu, Jianhong Lian

**Affiliations:** 1 Department of Thoracic Surgery, Shanxi Province Cancer Hospital/Shanxi Hospital Affiliated to Cancer Hospital, Chinese Academy of Medical Sciences/Cancer Hospital Affiliated to Shanxi Medical University, Taiyuan, Shanxi, China; 2 Department of General Surgery, Shanxi Bethune Hospital, Shanxi Academy of Medical Sciences, Tongji Shanxi Hospital, Third Hospital of Shanxi Medical University, Taiyuan, Shanxi, China; 3 Department of Anesthesiology, Shanxi Province Cancer Hospital/Shanxi Hospital Affiliated to Cancer Hospital, Chinese Academy of Medical Sciences/Cancer Hospital Affiliated to Shanxi Medical University, Taiyuan, Shanxi, China

**Keywords:** Recurrent laryngeal nerve, Esophagectomy, Esophageal squamous cell carcinoma, Lymphadenectomy, Survival

## Abstract

**Purpose:**

The clinical relevance of recurrent laryngeal nerve lymph node (RLN LN) management in early-stage resectable esophageal squamous cell carcinoma (ESCC) remains controversial. We evaluated the association of RLN LN retrieval with overall survival (OS) and disease-free survival (DFS) in patients with pT1bN0/pT2N0 thoracic ESCC and examined whether total lymph node (LN) yield was associated with outcomes.

**Methods:**

We retrospectively analyzed 212 patients with pT1bN0 or pT2N0 ESCC who underwent esophagectomy between January 2015 and September 2018. Patients were categorized by RLN LN retrieval status (no RLN LN retrieved *vs* ≥1 RLN LN retrieved (sampling or dissection)) and by total LN yield (<15 *vs* ≥15). RLN LN procedures were further classified as sampling *vs* formal dissection and unilateral *vs* bilateral retrieval. OS and DFS were estimated using Kaplan–Meier methods and compared with log-rank tests. To reduce confounding, we applied stabilized inverse probability of treatment weighting (sIPTW) based on prespecified preoperative covariates (excluding postoperative variables), with weight trimming at the 1st–99th percentiles, and fitted weighted Cox models with robust standard errors. Sensitivity analyses additionally adjusted for postoperative factors and included an approach-restricted analysis within the McKeown subgroup.

**Results:**

RLN LN retrieval (≥1) was associated with improved OS and DFS *vs* no RLN LN retrieved in both unweighted and sIPTW-weighted analyses (sIPTW-weighted HR 2.75, 95% CI [1.53–4.96] for OS; HR 2.84, 95% CI [1.61–5.01] for DFS; both *p* < 0.001). In sIPTW-adjusted multivariable models, absence of RLN LN retrieval remained associated with higher risks of death (adjusted HR 3.18, 95% CI [1.60–6.30]; *p* < 0.001) and recurrence/death (adjusted HR 3.51, 95% CI [1.86–6.63]; *p* < 0.001). Recurrent laryngeal nerve palsy occurred more frequently in the RLN LN retrieval group (21.9% *vs* 9.3%, *p* = 0.021). In exploratory procedure-focused analyses among RLN LN–retrieved patients, formal dissection (*vs* sampling) was associated with improved DFS (HR 0.40, 95% CI [0.18–0.90]; *p* = 0.027) with a borderline OS signal, whereas bilateral retrieval showed no additional benefit. Total LN yield (<15 *vs* ≥15) was not materially associated with OS or DFS overall or within pT1b/pT2 strata.

**Conclusions:**

In patients with pT1bN0/pT2N0 thoracic ESCC, retrieval of at least one RLN LN was associated with improved OS and DFS, whereas higher total LN yield was not clearly associated with survival. Because RLN-chain nodal retrieval was closely related to surgical approach and lymphadenectomy field, these findings should be interpreted as observational associations rather than proof of an independent causal benefit of RLN LN dissection itself. Larger, ideally multicenter studies are needed to clarify the respective contributions of RLN-chain nodal management, staging adequacy, and operative approach to long-term outcomes.

## Introduction

According to the GLOBOCAN 2022 statistics, esophageal cancer ranked 11th in incidence and 7th in mortality worldwide in 2022 (Bray et al., 2024). Notably, China is a major contributor to the global burden of esophageal cancer. In China, esophageal cancer ranks 7th in incidence and 4th in mortality (Ji et al., 2024). Globally, the histological types of esophageal cancer primarily include adenocarcinoma and squamous cell carcinoma (SCC). Esophageal adenocarcinoma is the predominant pathological type in North America and Europe, while in China, esophageal squamous cell carcinoma (ESCC) accounts for over 90% of cases (Liang, Fan & Qiao, 2017).

With advances in endoscopic techniques, an increasing proportion of patients with early or superficial esophageal lesions are being treated with endoscopic mucosal resection (Nihei et al., 2023). However, for T1bN0 or T2N0 patients, surgery remains the preferred treatment method (Smyth et al., 2017; Udagawa, Ueno & Tsutsumi, 2007). Even in patients with clinical T1N0 disease, the risk of lymph node metastasis remains non-negligible, and solitary extrathoracic nodal metastasis is not uncommon; therefore, systematic lymphadenectomy should not be omitted (Akutsu et al., 2016; Shimada et al., 2006).

Esophageal cancer frequently metastasizes through the lymphatic system, making the evaluation of lymph node (LN) metastasis critically important (Bogoevski et al., 2008). According to the research by Zhang et al. (2022), the recurrent laryngeal nerve lymph nodes (RLN LN) have the highest rate of metastasis in thoracic squamous cell carcinoma, with reported metastasis rates ranging from 19.4% to 41.9% (Udagawa et al., 2012; Li et al., 2012). Even for early-stage ESCC, RLN LN may harbor occult metastases (Tachimori et al., 2011). Hong et al. (2022) reported an association between more extensive RLN LN management and lower recurrence risk in early-stage ESCC. However, due to the narrow space in which the RLN LN are located and the potential for nerve damage, dissection of the RLN LN poses a significant challenge for thoracic surgeons.

The optimal extent of lymphadenectomy in surgery for esophageal cancer remains controversial (Vagliasindi et al., 2023). Previous studies have suggested that management of the RLN LN basin may be clinically relevant in early-stage ESCC, although the extent to which this reflects therapeutic clearance, staging adequacy, or differences in surgical approach remains uncertain (Yu et al., 2016). In addition, the prognostic relevance of total lymph node yield in pN0 ESCC remains debated (Li et al., 2023). Therefore, in this study, we examined the association of RLN-chain nodal retrieval with OS and DFS in patients with pT1bN0 and pT2N0 thoracic ESCC, while also assessing whether total LN yield was associated with survival.

## Methods

Data were obtained from our hospital database. We retrospectively analyzed patients with ESCC classified as pT1bN0 or pT2N0 from January 2015 to September 2018. This study was approved by the Ethics Committee of Shanxi Provincial Cancer Hospital, Taiyuan, China on 31 August 2024 (KY2024130). Consent was waived by the Ethics Committee of Shanxi Provincial Cancer Hospital due to the retrospective nature of the study.

We retrieved demographic and pathological information, including sex, age, body mass index (BMI), hypertension, diabetes, smoking, and alcohol consumption. Tumor characteristics such as location (upper, middle, or lower thoracic), surgical method (McKeown, Ivor Lewis, or Sweet), tumor length, T stage, N stage, number of LN dissected, number of RLN LN resected, whether receiving adjuvant treatment and postoperative complications (recurrent laryngeal nerve palsy, anastomotic leakage and respiratory complications) were also recorded. Respiratory complications include pneumonia, pleural effusion requiring paracentesis or closed chest tube drainage, or pneumothorax. For patients diagnosed before 2018, we re-staged them using the latest 8th edition TNM staging system for esophageal cancer from the American Joint Committee on Cancer (AJCC). Follow-up was conducted every 3 months during the first year and every 6 months thereafter. The inclusion criteria were as follows: (1) esophagectomy was performed; (2) tumor located in the thoracic esophagus; (3) pathological results confirmed squamous cell carcinoma; (4) pathological results confirmed pT1bN0 or pT2N0. The exclusion criteria were as follows: (1) presence of other malignant tumors; (2) previous neoadjuvant therapy; (3) pathological results showed non-squamous cell carcinoma; (4) pathological stage was Tis/T1a/T3/T4/M1; (5) required data were missing.

First, patients were divided into two groups according to RLN LN retrieval status: no RLN LN retrieved *vs* ≥1 RLN LN retrieved (including sampling or dissection). The no-RLN-retrieval group consisted of patients in whom RLN LN dissection was either not attempted or attempted but yielded no RLN LN. RLN LN sampling was defined as removal of a single RLN LN on one side or one node from each side, whereas formal RLN LN dissection was defined as retrieval of more than one node from at least one side. Second, patients were divided into two groups according to the number of dissected LN (LN < 15 and ≥ 15), with the cutoff of 15 nodes defined on the basis of the NCCN guidelines for esophageal cancer (Ajani et al., 2023). The surgical approach was determined by experienced thoracic surgeons based on preoperative assessments, including contrast-enhanced computed tomography (CT), LN ultrasonography, and endoscopic ultrasonography. Subsequently, statistical analyses were conducted for each group. OS was defined as the time from surgery to death from any cause or last follow-up, and DFS was defined as the time from surgery to recurrence, death, or last follow-up, whichever occurred first.

Categorical variables were expressed as percentages, while continuous variables were expressed as means or medians. We calculated the results using the χ^2^ test or Fisher’s exact test. For continuous variables, results were presented as the mean with the standard deviation (SD), T-tests were used to compare the differences between groups. Differences in OS and DFS between groups were assessed using log-rank analysis, and survival curves were generated using the Kaplan-Meier method. Univariate and multivariable Cox proportional hazards regression analyses were performed to identify independent predictors associated with survival. To mitigate confounding in comparisons between RLN LN retrieval groups, we applied stabilized inverse probability of treatment weighting (sIPTW). Propensity scores were estimated using logistic regression with prespecified preoperative covariates; postoperative variables were not included in the propensity score model. Stabilized weights were trimmed at the 1st and 99th percentiles. Covariate balance before and after weighting was assessed using standardized mean differences (SMD) and visualized with a Love plot. Weighted Cox models used robust standard errors. In addition, a sensitivity analysis was performed by further adjusting the survival models for postoperative factors (recurrent laryngeal nerve palsy, anastomotic leakage, respiratory complications, and receipt of adjuvant therapy). Because RLN-chain nodal retrieval was closely related to surgical approach, we additionally performed an approach-restricted sensitivity analysis within the McKeown subgroup to reduce operative heterogeneity. Statistical analyses were conducted using R software (version 4.4.1). A two-sided *P* value < 0.05 was considered statistically significant. We conducted a complete-case analysis. Patients with missing values in covariates required for propensity score estimation and/or survival analyses were excluded from the analytic cohort.

## Results

### Patient characteristics

A total of 212 patients were included, of whom 137 (64.6%) were classified as having RLN LN retrieval (≥1 node) and 75 (35.4%) as having no RLN LN retrieval. Baseline characteristics by RLN LN retrieval status are summarized in Table 1. In the unweighted cohort, most demographic and comorbidity variables were similar between groups, whereas clinically meaningful imbalance was observed for surgical approach and several tumor-related variables as reflected by SMD. After applying sIPTW with trimming at the 1st–99th percentiles, overall covariate balance improved and most covariates achieved small residual imbalance; balance diagnostics are shown in the Love plot (Fig. S1).

**Table 1:** Baseline characteristics before and after stabilized inverse probability of treatment weighting.

Variables	RLN LN retrieval ≥ 1 (*n* = 137)	RLN LN retrieval = 0 (*n* = 75)	Unweighted SMD	sIPTW-weighted SMD
Age, *n* (%)			0.021	0.079
<65	90 (65.69)	50 (66.67)		
≥65	47 (34.31)	25 (33.33)		
BMI, *n* (%)			0.051	0.074
<24	91 (66.42)	48 (64.00)		
≥24	46 (33.58)	27 (36.00)		
Sex, *n* (%)			0.045	0.002
Female	67 (48.91)	35 (46.67)		
Male	70 (51.09)	40 (53.33)		
Hypertension, *n* (%)			0.066	0.098
No	104 (75.91)	59 (78.67)		
Yes	33 (24.09)	16 (21.33)		
Diabetes, *n* (%)			0.137	0.019
No	132 (96.35)	70 (93.33)		
Yes	5 (3.65)	5 (6.67)		
Cardiovascular diseases, *n* (%)			0.065	0.111
No	134 (97.81)	74 (98.67)		
Yes	3 (2.19)	1 (1.33)		
Smoking history, *n* (%)			0.084	0.070
No	77 (56.20)	39 (52.00)		
Yes	60 (43.80)	36 (48.00)		
Alcohol consumption, *n* (%)			0.122	0.098
No	99 (72.26)	50 (66.67)		
Yes	38 (27.74)	25 (33.33)		
Tumor location, *n* (%)			0.168	0.064
Lower	31 (22.63)	16 (21.33)		
Middle	88 (64.23)	53 (70.67)		
Upper	18 (13.14)	6 (8.00)		
Tumor length, *n* (%)			0.058	0.037
<3	55 (40.15)	28 (37.33)		
≥3	82 (59.85)	47 (62.67)		
Operation, *n* (%)			1.426	0.612
Ivor Lewis	10 (7.30)	16 (21.33)		
McKeown	127 (92.70)	28 (37.33)		
Sweet	0 (0.00)	31 (41.33)		
cT stage, *n* (%)			0.164	0.101
1	43 (31.39)	23 (30.67)		
2	94 (68.61)	51 (68.00)		
3	0 (0.00)	1 (1.33)		
cN stage, *n* (%)			0.055	0.069
0	120 (87.59)	67 (89.33)		
1	17 (12.41)	8 (10.67)		

**Notes:**

Abbreviations: RLN, recurrent laryngeal nerve; BMI, body mass index; SMD, standardized mean difference; sIPTW, stabilized inverse probability of treatment weighting.

Propensity scores were estimated using logistic regression with prespecified covariates. Stabilized IPTW weights were applied and trimmed at the 1st and 99th percentiles. *P* values are omitted; balance is assessed using SMD.

Pathologic stage distribution and key postoperative outcomes are presented in Table 2. The proportions of pT1b *vs* pT2 disease were comparable between groups (*p* = 0.488). Among postoperative events, recurrent laryngeal nerve palsy occurred more frequently in the RLN LN retrieval group (21.9% *vs* 9.3%, *p* = 0.021), whereas rates of anastomotic leakage (21.9% *vs* 29.3%, *p* = 0.229), respiratory complications (38.0% *vs* 41.3%, *p* = 0.630), and receipt of adjuvant therapy (28.5% *vs* 30.7%, *p* = 0.736) did not differ significantly.

**Table 2:** Pathologic stage and postoperative outcomes by RLN LN retrieval.

Variables	RLN LN retrieval ≥ 1 (*n* = 137)	RLN LN retrieval = 0 (*n* = 75)	*P* value
pT stage, *n* (%)			0.488
pT1b	50 (36.50)	31 (41.33)	
pT2	87 (63.50)	44 (58.67)	
LN dissection, *n* (%)			<0.001
<15	47 (34.31)	52 (69.33)	
≥15	90 (65.69)	23 (30.67)	
MIE, *n* (%)			0.079
No	42 (30.65)	32 (42.67)	
Yes	95 (69.35)	43 (57.33)	
T stage migration, *n* (%)			0.593
No	113 (82.48)	64 (85.33)	
Yes	24 (17.52)	11 (14.67)	
N stage migration, *n* (%)			0.707
No	120 (87.59)	67 (89.33)	
Yes	17 (12.41)	8 (10.67)	
RLN palsy, *n* (%)			0.021
No	107 (78.10)	68 (90.67)	
Yes	30 (21.90)	7 (9.33)	
Anastomotic leakage, *n* (%)			0.229
No	107 (78.10)	53 (70.67)	
Yes	30 (21.90)	22 (29.33)	
Respiratory complications, *n* (%)			0.630
No	85 (62.04)	44 (58.67)	
Yes	52 (37.96)	31 (41.33)	
Received adjuvant therapy, *n* (%)			0.736
No	98 (71.53)	52 (69.33)	
Yes	39 (28.47)	23 (30.67)	

**Notes:**

Abbreviations: RLN, recurrent laryngeal nerve; MIE, minimally invasive esophagectomy. Values are presented as *n* (%).

*P* values were calculated using Pearson’s chi-square test or Fisher’s exact test as appropriate.

### Association of RLN LN retrieval with OS and DFS

**Figure 1:**
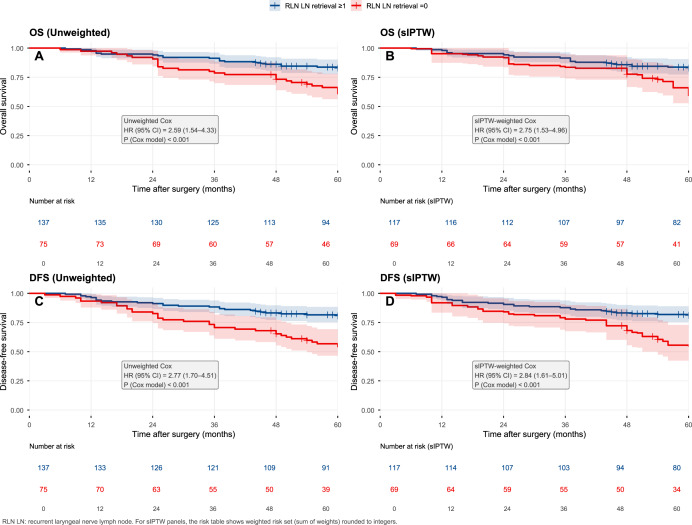
Kaplan–Meier curves for OS and DFS before and after sIPTW. Kaplan–Meier curves comparing overall survival (OS) and disease-free survival (DFS) between patients with RLN LN retrieval ≥1 and RLN LN retrieval = 0. Panels show unweighted analyses (A, C) and sIPTW-weighted analyses (B, D). Hazard ratios (HRs) and *P* values are from Cox proportional hazards models.

In Kaplan–Meier analyses, RLN LN retrieval (≥1) was associated with improved survival compared with no retrieval. In the unweighted Cox model, the no-retrieval group had significantly worse OS (HR 2.59, 95% CI [1.54–4.33]; *p* < 0.001) and worse DFS (HR 2.77, 95% CI [1.70–4.51]; *p* < 0.001). These associations remained after sIPTW adjustment, with HR 2.75 (95% CI [1.53–4.96]; *p* < 0.001) for OS and HR 2.84 (95% CI [1.61–5.01]; *p* < 0.001) for DFS (Fig. 1).

### Multivariable sIPTW-adjusted Cox models

**Figure 2:**
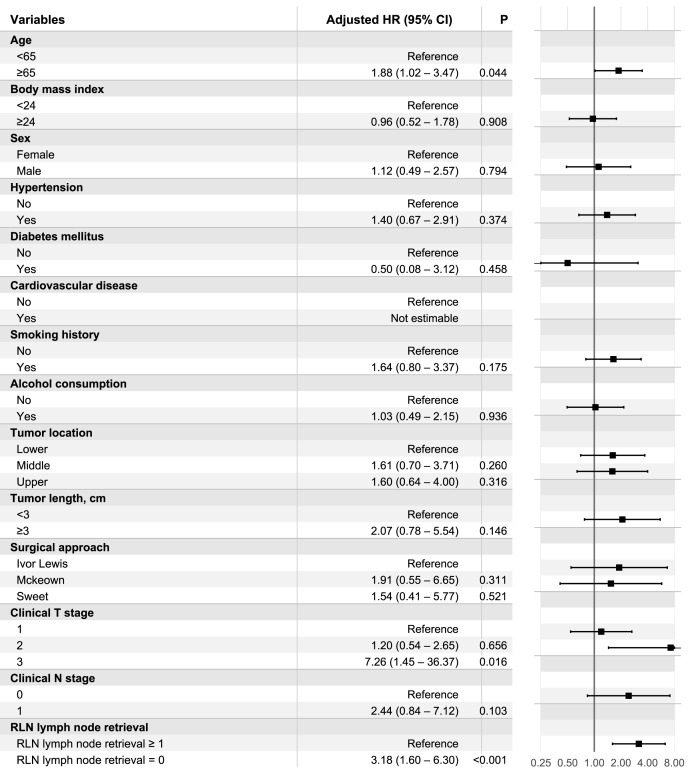
sIPTW-adjusted multivariable Cox model for OS. Forest plot of sIPTW-adjusted multivariable Cox proportional hazards model for OS with robust standard errors. Adjusted HRs with 95% confidence intervals are presented for prespecified covariates, including RLN LN retrieval status.

In sIPTW-adjusted multivariable Cox models with robust standard errors, RLN LN retrieval remained independently associated with outcomes. For OS, absence of RLN LN retrieval was associated with higher mortality risk (adjusted HR 3.18, 95% CI [1.60–6.30]; *p* < 0.001) (Fig. 2). Older age (≥65 years) was also associated with worse OS (adjusted HR 1.88, 95% CI [1.02–3.47]; *p* = 0.044), and advanced clinical T stage (cT3 *vs* cT1) showed an increased hazard (adjusted HR 7.26, 95% CI [1.45–36.37]; *p* = 0.016).

**Figure 3:**
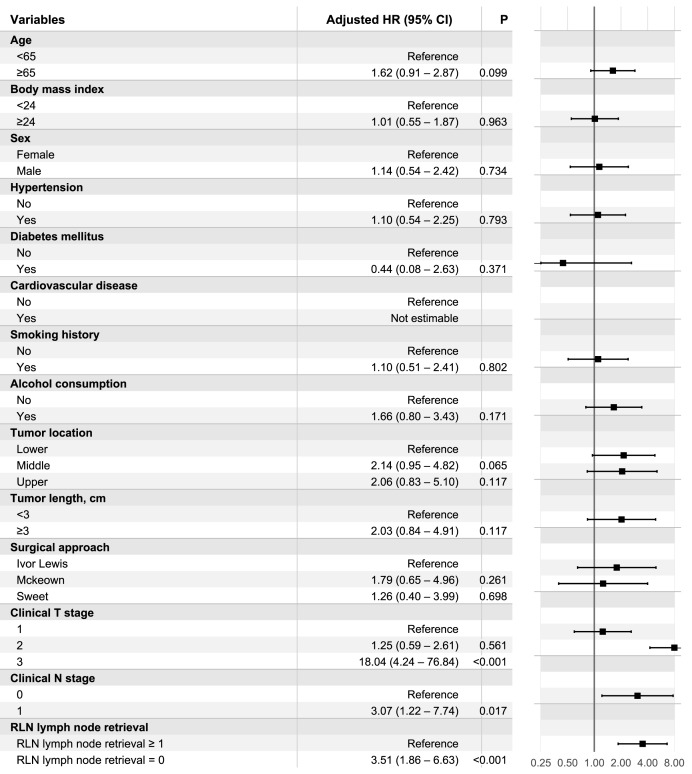
sIPTW-adjusted multivariable Cox model for DFS. Forest plot of sIPTW-adjusted multivariable Cox proportional hazards model for DFS with robust standard errors. Adjusted HRs with 95% confidence intervals are presented for prespecified covariates, including RLN LN retrieval status.

For DFS, no RLN LN retrieval was similarly associated with increased recurrence risk (adjusted HR 3.51, 95% CI [1.86–6.63]; *p* < 0.001) (Fig. 3). Clinical N1 disease (*vs* cN0) was associated with worse DFS (adjusted HR 3.07, 95% CI [1.22–7.74]; *p* = 0.017), and cT3 disease was strongly associated with poorer DFS (adjusted HR 18.04, 95% CI [4.24–76.84]; *p* < 0.001).

Results were directionally consistent in a sensitivity analysis that additionally adjusted for postoperative factors (Figs. S2, S3).

### Sensitivity analysis restricted to McKeown esophagectomy

**Figure 4:**
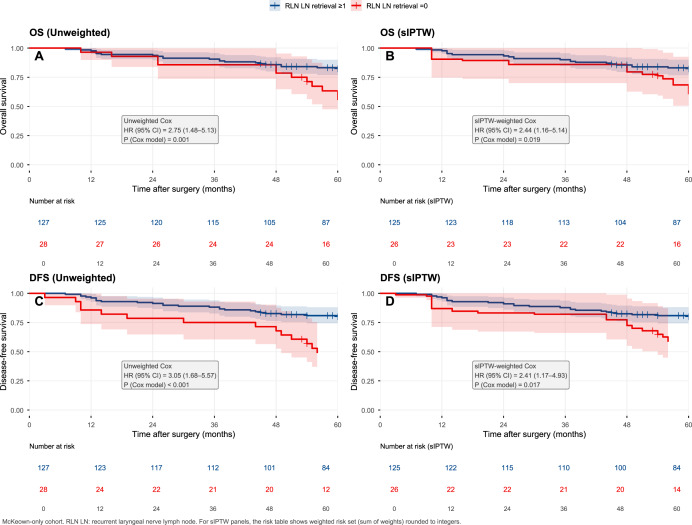
Sensitivity analysis restricted to McKeown esophagectomy. Unweighted and sIPTW-weighted Kaplan–Meier curves for overall survival (OS) and disease-free survival (DFS) comparing patients with RLN LN retrieval ≥1 *vs* RLN LN retrieval = 0 among those undergoing McKeown esophagectomy only. (A) and (B) show OS; (C) and (D) show DFS. Hazard ratios (HRs) and two-sided *P* values are from unweighted or sIPTW-weighted Cox models with robust standard errors. In sIPTW panels, numbers at risk represent weighted risk sets (sum of weights), rounded to integers.

Given the strong relationship between RLN-chain nodal retrieval and surgical approach, we performed an additional sensitivity analysis restricted to patients undergoing McKeown esophagectomy. Within this more homogeneous operative subgroup, absence of RLN LN retrieval remained associated with worse survival in both unweighted and sIPTW-weighted analyses (Fig. 4). In unweighted analyses, the hazard ratios were 2.75 (95% CI [1.48–5.13]; *p* = 0.001) for OS and 3.05 (95% CI [1.68–5.57]; *p* < 0.001) for DFS. After sIPTW adjustment, the associations remained directionally consistent and statistically significant, with HR 2.44 (95% CI [1.16–5.14]; *p* = 0.019) for OS and HR 2.41 (95% CI [1.17–4.93]; *p* = 0.017) for DFS. These results reduce concern that the primary findings were entirely driven by cross-approach differences, but they do not eliminate the possibility of residual confounding related to surgeon-level or intraoperative factors. Because no patient undergoing Sweet esophagectomy had RLN LN retrieval in the final analytic dataset, a meaningful within-Sweet stratified analysis was not feasible.

### Procedure-related subgroup analyses

Among patients who underwent RLN LN retrieval, bilateral retrieval did not confer additional benefit over unilateral retrieval in sIPTW-weighted analyses for either OS (HR 0.97, 95% CI [0.39–2.40]; *p* = 0.950) or DFS (HR 0.99, 95% CI [0.42–2.36]; *p* = 0.988); however, the estimates were imprecise with wide confidence intervals, reflecting limited events (Figs. S4A, S4B). Formal RLN LN dissection was associated with a lower hazard of recurrence compared with sampling in weighted analyses (DFS HR 0.40, 95% CI [0.18–0.90]; *p* = 0.027), while the OS association was borderline (HR 0.43, 95% CI [0.19–1.01]; *p* = 0.053); these procedure-focused comparisons should be interpreted as exploratory given the limited number of events (Figs. S4C, S4D). Similar associations were observed in the unweighted Kaplan–Meier analyses (Fig. S5).

### Total lymph node yield

**Figure 5:**
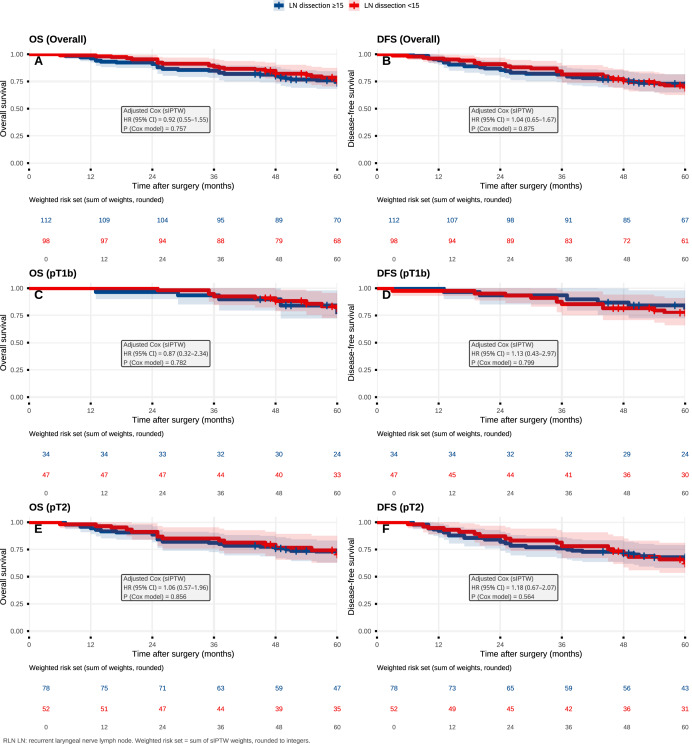
Total lymph node yield (<15 *vs* ≥15) and survival (sIPTW-weighted). sIPTW-weighted Kaplan–Meier curves comparing OS and DFS between patients with <15 *vs* ≥15 total lymph nodes examined, shown in the overall cohort (A, B) and stratified by pathologic T stage (pT1b: C, D; pT2: E, F). HRs and *P* values are from sIPTW-weighted Cox models with robust standard errors.

Total lymph node yield (<15 *vs* ≥15) was not materially associated with OS or DFS in either unweighted or sIPTW-weighted analyses, in the overall cohort or after stratification by pathologic T stage (Figs. 5 and S6).

## Discussion

In this retrospective cohort of patients with early-stage resectable ESCC staged as pT1bN0 or pT2N0, retrieval of at least one recurrent laryngeal nerve lymph node (RLN LN) was associated with improved OS and DFS. This association remained directionally consistent after sIPTW adjustment and multivariable Cox modeling. However, these findings should not be interpreted as definitive proof of an independent causal benefit of RLN LN dissection itself. RLN-chain nodal retrieval was closely related to surgical approach and the extent of upper mediastinal lymphadenectomy, and therefore the observed survival differences may also reflect differences in nodal clearance, staging adequacy, and other unmeasured aspects of operative decision-making. In particular, because Sweet esophagectomy may limit access to the RLN chain, whereas McKeown esophagectomy more often permits systematic upper mediastinal nodal dissection, part of the observed association may reflect approach-related differences in oncologic clearance rather than an isolated effect of RLN LN retrieval alone. To further address this concern, we performed an additional sensitivity analysis restricted to patients undergoing McKeown esophagectomy. Within this more homogeneous operative subgroup, absence of RLN LN retrieval remained associated with worse OS and DFS in both unweighted and sIPTW-weighted analyses, suggesting that the main findings were not solely driven by between-approach differences, although residual confounding cannot be excluded. Because our primary exposure was defined as retrieval of at least one RLN lymph node, the present findings should not be interpreted as direct proof that minimal node removal itself is therapeutic; rather, they are more appropriately viewed as observational evidence related to RLN-chain nodal management, clearance, and staging adequacy.

For patients with N0 ESCC, there is ongoing controversy regarding the optimal number of LN to be dissected. Xu et al. (2023) showed that for patients with stage N0 disease, dissecting ≤15 LNs was associated with increased mortality compared with dissecting >15 LNs. However, in our study, there was no significant difference in OS or DFS between patients with <15 and ≥15 total LNs retrieved. One possible explanation is the difference in study populations, as prior database-based analyses included a substantial proportion of adenocarcinoma, whereas our cohort was restricted to ESCC. A study by Li et al. (2023) focusing on ESCC in mainland China reached a conclusion similar to ours: in patients with N0 ESCC, increasing total LN yield did not translate into a clear survival benefit. Taken together, these findings suggest that total LN yield alone may be an imperfect surrogate for oncologic adequacy in early-stage ESCC, particularly when the dissection field differs across patients.

The prognosis of early-stage ESCC remains unsatisfactory, as the recurrence rate after radical resection in patients with pathological N0 disease may still be substantial (Guo et al., 2014). One biologically plausible explanation is the characteristic longitudinal lymphatic drainage of the esophagus. The submucosal plexus can directly connect to regional lymphatic pathways, and it has been repeatedly shown that submucosal lymphatic flow drains into the RLN LN basin (Wang et al., 2018). Even in early-stage ESCC, cancer cells may therefore spread along the recurrent laryngeal nerve to the upper mediastinal lymphatic chain, including the RLN LN (Tachimori, 2017).

From a surgical perspective, extensive RLN LN resection may increase the risk of complications, such as RLN palsy and aspiration-induced pneumonia (Kuo et al., 2024). The recurrent laryngeal nerve is susceptible to various injuries associated with resection, including inadequate blood supply to the nerve, thermal injury, improper ligation, contusion, and traction (Taniyama et al., 2015). Given the higher rate of RLN palsy observed in the RLN LN retrieval group, the potential oncologic benefit should be weighed against functional morbidity. To mitigate nerve injury, intraoperative recurrent laryngeal nerve monitoring and meticulous nerve-sparing dissection with minimized traction and thermal spread may help reduce RLN palsy while preserving oncologic clearance (Wu et al., 2024; Scholtemeijer et al., 2017). Hong et al. (2022) studied 567 patients with pT1N0 disease and 927 patients with cT1N0 disease and reported that the optimal number of RLN LNs to resect was four. We were unable to define an optimal RLN LN count in the present study, likely because of the smaller sample size and lower number of events, but also because our cohort was defined by pathological stage after surgery rather than by a preoperative clinical stage framework.

Beyond procedure-specific nerve injury, surgery also triggers a systemic inflammatory response, including postoperative rises in IL-6 and CRP, and higher early postoperative IL-6 levels have been associated with an increased risk of major complications after minimally invasive esophagectomy (Rettig et al., 2016). Surgery-related inflammatory stress is also accompanied by transient postoperative immunosuppression, which may impair anti-tumor immune surveillance and potentially facilitate recurrence or metastasis (Tang et al., 2020). These biological considerations provide a rationale for caution when interpreting more extensive nodal dissection as uniformly beneficial, particularly when the balance between improved nodal clearance and added operative stress may vary across surgical approaches and individual patients.

Tumor length may correlate with invasion depth and overall tumor burden, but evidence suggests that lymph node metastasis in superficial esophageal cancer is driven primarily by depth of invasion rather than length, particularly in T1 disease. In fact, even widely spreading superficial lesions confined to T1a (especially EP/LPM) have an almost negligible risk of nodal metastasis, whereas deeper invasion is associated with a substantially higher likelihood of nodal involvement (Shimada et al., 2006; Kitagawa et al., 2023). Therefore, we refocused our interpretation on depth-based risk stratification rather than tumor length when discussing nodal metastasis and surgical decision-making.

Since our study was not designed to delineate causal mechanisms, further research is warranted. One plausible explanation is pathologic understaging in a subset of patients, whereby occult nodal disease was not detected and patients were therefore misclassified as pN0. Prior studies have suggested that more extensive lymph node assessment may increase the likelihood of postoperative upstaging (Shridhar et al., 2013), which is consistent with this possibility. Another explanation is that micrometastatic disease may have been present in some RLN LNs despite a conventional pN0 classification. Yang et al. (2024) found that pN0 ESCC patients with LN micrometastasis had worse prognosis, which also supports this interpretation. A particularly important alternative explanation is stage migration. Because the lymphadenectomy field differed between groups, more systematic RLN-chain nodal assessment may have improved the accuracy of pN0 classification in the RLN LN–retrieved group by identifying occult nodal disease that would otherwise have remained unrecognized. This interpretation is also consistent with approach-related constraints, particularly the limited feasibility of RLN-chain dissection during Sweet esophagectomy, which may influence both the oncologic adequacy of nodal staging and the extent of upper mediastinal clearance. Therefore, the observed survival association may reflect a combination of therapeutic clearance, more accurate staging, and residual differences in operative strategy. Consequently, part of the observed survival difference may reflect more accurate nodal staging rather than a purely therapeutic effect of RLN-chain nodal retrieval itself. Although the additional McKeown-only sensitivity analysis reduced concern that the main results were entirely driven by cross-approach differences, it does not eliminate the possibility of residual confounding related to surgeon preference, intraoperative judgment, or other unmeasured factors.

In our analyses, total LN yield was not clearly associated with survival, whereas RLN LN retrieval showed a consistent association with OS and DFS. One possible explanation is the disproportionately high metastatic involvement of the RLN nodal basin in thoracic ESCC relative to some other nodal stations (Yang et al., 2022). Accordingly, the completeness and field of lymphadenectomy may be more clinically relevant than the total number of nodes retrieved alone. Importantly, LN yield is an observed result influenced not only by surgical extent but also by patient anatomy and pathological processing; therefore, it should not be interpreted as a surgical target in itself. In this setting, the present findings may indicate that addressing the RLN-chain nodal basin is more informative than total node count alone, but they should not be taken as direct proof that retrieval of a minimal number of RLN nodes has an isolated therapeutic effect.

Several limitations of this study should be acknowledged. First, this was a single-center retrospective study, and patient selection, operative strategy, and pathological processing at our institution may not be fully representative of other settings. Second, RLN-chain nodal retrieval was strongly associated with surgical approach, and the observational design does not allow complete separation of approach effects from nodal management itself. Although we adjusted for measured covariates using sIPTW and multivariable modeling and additionally performed a McKeown-only sensitivity analysis, residual confounding from unmeasured factors, such as surgeon preference, technical expertise, and intraoperative findings, cannot be excluded. Third, because the number of outcome events was limited relative to the number of covariates, subgroup and procedure-focused analyses should be interpreted as exploratory and hypothesis-generating rather than definitive. Fourth, differences in lymphadenectomy field may have introduced stage migration, such that part of the observed survival association may reflect improved staging adequacy rather than a purely therapeutic effect. Accordingly, our findings are best interpreted as real-world observational evidence relevant to RLN-chain nodal management and staging adequacy, and they require confirmation in larger, ideally multicenter studies conducted in more homogeneous surgical settings. Notably, no patient in the Sweet subgroup underwent RLN LN retrieval in the final analytic dataset; therefore, no meaningful within-Sweet comparison was possible, and the Sweet subgroup mainly contributes to the interpretation of approach-related heterogeneity rather than to subgroup-specific causal inference.

## Conclusions

In this single-center retrospective cohort of patients with early-stage resectable ESCC (pT1bN0/pT2N0), retrieval of at least one recurrent laryngeal nerve lymph node was associated with improved OS and DFS, whereas higher total lymph node yield was not clearly associated with survival. However, because RLN-chain nodal retrieval was closely related to surgical approach and lymphadenectomy field, these findings should be interpreted as observational associations rather than proof of an independent causal benefit of RLN lymph node dissection itself. Larger, ideally multicenter studies conducted in more homogeneous surgical settings are needed to clarify the respective contributions of RLN-chain nodal management, staging adequacy, and operative approach to long-term outcomes, while also balancing potential oncologic benefit against perioperative morbidity.

## Supplemental Information

10.7717/peerj.21293/supp-1 Supplemental Information 1 Covariate balance before and after weighting. Covariate balance (standardized mean differences, SMD) between the RLN LN resection ≥1 group and the RLN LN resection =0 group, before weighting (unadjusted) and after stabilized inverse probability of treatment weighting (sIPTW) with trimming at the 1st–99th percentiles.

10.7717/peerj.21293/supp-2 Supplemental Information 2 Sensitivity analysis for overall survival with additional adjustment for postoperative factors. Forest plot of Cox proportional hazards model for overall survival (OS) in a sensitivity analysis that additionally adjusted for postoperative factors, including recurrent laryngeal nerve palsy, anastomotic leakage, respiratory complications, and receipt of adjuvant therapy. Hazard ratios (HRs) with 95% confidence intervals (CIs) are presented for RLN LN resection status and covariates included in the model.

10.7717/peerj.21293/supp-3 Supplemental Information 3 Sensitivity analysis for disease-free survival with additional adjustment for postoperative factors. Forest plot of Cox proportional hazards model for disease-free survival (DFS) in a sensitivity analysis that additionally adjusted for postoperative factors, including recurrent laryngeal nerve palsy, anastomotic leakage, respiratory complications, and receipt of adjuvant therapy. Hazard ratios (HRs) with 95% confidence intervals (CIs) are presented for RLN LN resection status and covariates included in the model.

10.7717/peerj.21293/supp-4 Supplemental Information 4 Procedure-related subgroup analyses ( unweighted sIPTW-weighted KM ). Unweighted Procedure-related subgroup analyses using sIPTW-weighted Kaplan–Meier curves for procedure-related subgroup analyses curves. Panels A–B compare unilateral *versus* bilateral RLN LN resection retrieval; panels C–D compare RLN LN sampling *versus* formal RLN LN dissection. HRs and P values are from unadjusted sIPTW-weighted Cox models models with robust standard errors.

10.7717/peerj.21293/supp-5 Supplemental Information 5 Procedure-related subgroup analyses (unweighted). Unweighted Kaplan–Meier curves for procedure-related subgroup analyses. Panels A–B compare unilateral *versus* bilateral RLN LN retrieval; panels C–D compare RLN LN sampling *versus* formal RLN LN dissection. HRs and P values are from unadjusted Cox models.

10.7717/peerj.21293/supp-6 Supplemental Information 6 Total lymph node yield (<15 *vs* ≥15) and survival (unweighted). Unweighted Kaplan–Meier curves comparing OS and DFS between <15 *versus* ≥15 total lymph nodes examined, shown in the overall cohort (A–B) and stratified by pathologic T stage (pT1b: C–D; pT2: E–F). HRs and *P* values are from unadjusted Cox models.

10.7717/peerj.21293/supp-7 Supplemental Information 7 Raw data.

## Abbreviations

SCC: squamous cell carcinoma
ESCC: esophageal squamous cell carcinoma
RLN LN: recurrent laryngeal nerve lymph nodes
OS: overall survival
DFS: disease-free survival
LN: lymph nodes
SD: standard deviation
sIPTW: stabilized inverse probability of treatment weighting
SMD: standardized mean differences
BMI: body mass index
MIE: minimally invasive esophagectomy

## Additional Information and Declarations

### Data Availability

The following information was supplied regarding data availability:

The raw data is available in the Supplemental File.
