# Supplementary figures and images for: Association of recurrent laryngeal nerve lymph node retrieval with survival in early-stage resectable esophageal squamous cell carcinoma: a retrospective cohort study
Source: PeerJ. 2026 Jun 4;14:e21293. doi: 10.7717/peerj.21293 (PMC13242742; doi:10.7717/peerj.21293)

Covariate balance (SMD): Unweighted vs sIPTW (trimmed 1st–99th pct)

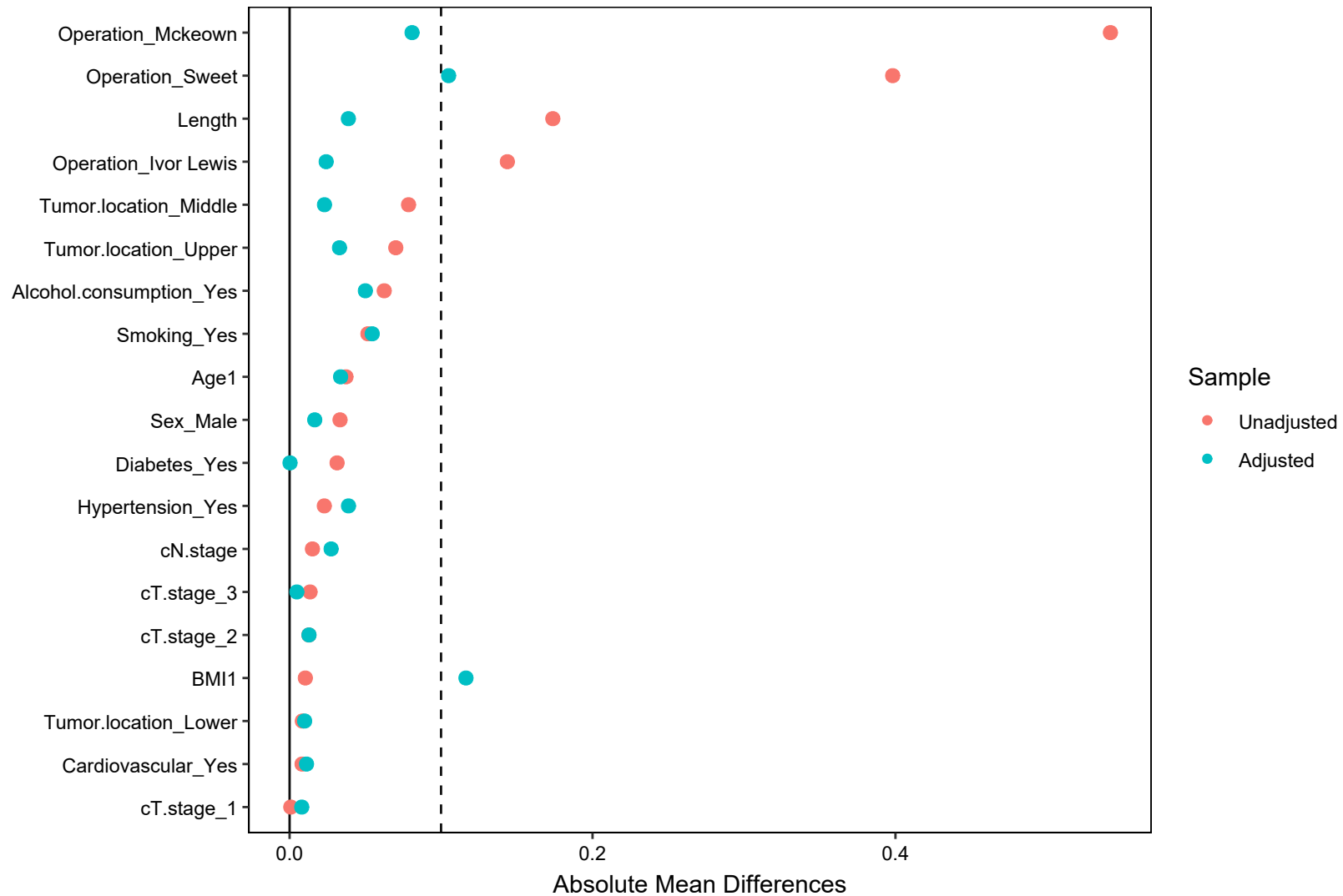

Supplement: Supplemental Information 1 — Covariate balance (standardized mean differences, SMD) between the RLN LN resection ≥1 group and the RLN LN resection =0 group, before weighting (unadjusted) and after stabilized inverse probability of treatment weighting (sIPTW) with trimming at the 1st–99th percentiles. [file peerj-14-21293-s001.pdf]

LN dissection ≥15    LN dissection <15

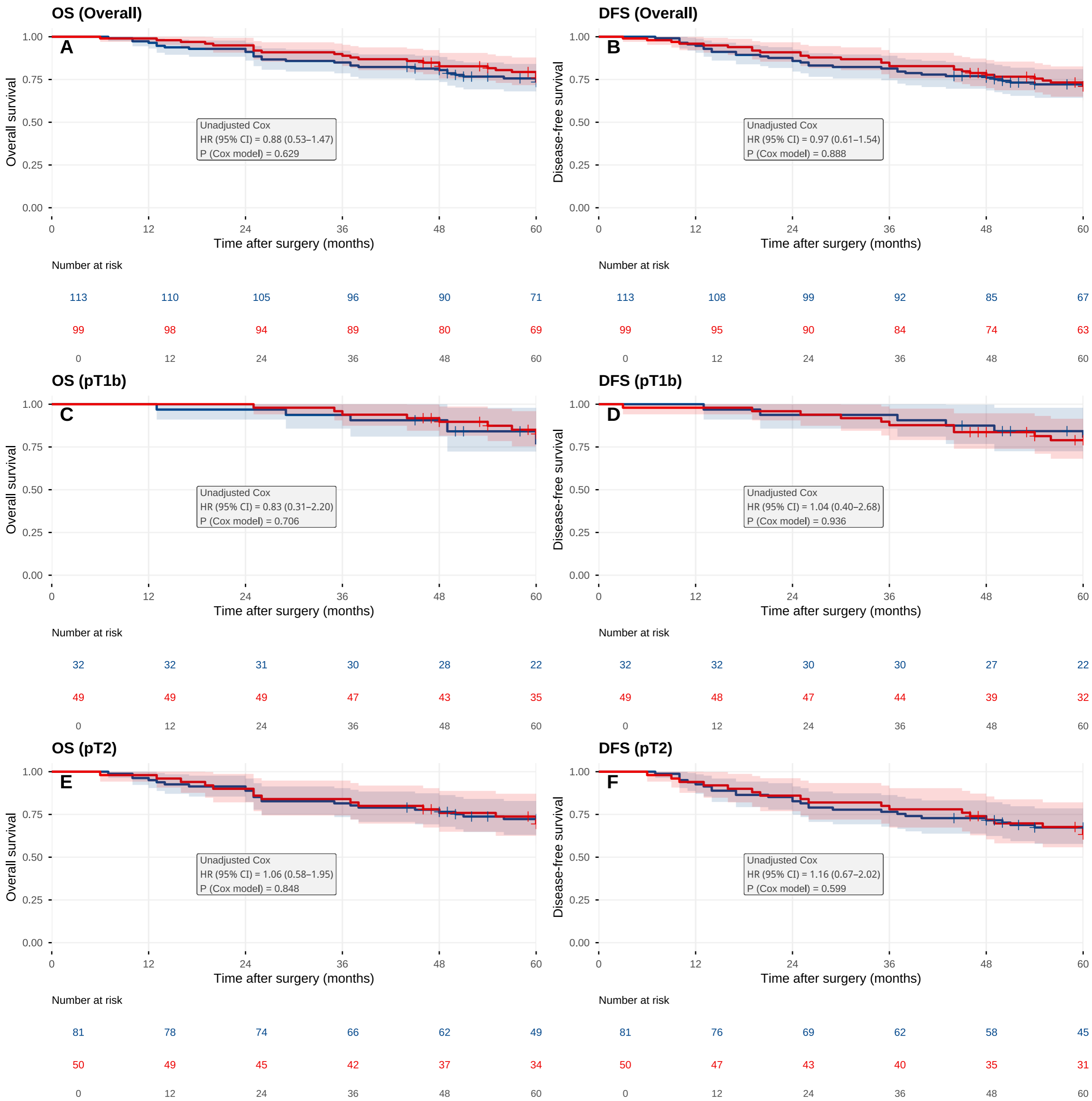

Supplement: Supplemental Information 6 — Unweighted Kaplan–Meier curves comparing OS and DFS between <15 versus ≥15 total lymph nodes examined, shown in the overall cohort (A–B) and stratified by pathologic T stage (pT1b: C–D; pT2: E–F). HRs and P values are from unadjusted Cox models. [file peerj-14-21293-s006.pdf]
