# Supplementary material for: Association of recurrent laryngeal nerve lymph node retrieval with survival in early-stage resectable esophageal squamous cell carcinoma: a retrospective cohort study
Source: PeerJ. 2026 Jun 4;14:e21293. doi: 10.7717/peerj.21293 (PMC13242742; doi:10.7717/peerj.21293)

Variables	Adjusted HR (95% CI)	P
**Age**		
<65	Reference	
≥65	2.05 (1.09 – 3.85)	0.025
**Body mass index**		
<24	Reference	
≥24	1.02 (0.55 – 1.88)	0.945
**Sex**		
Female	Reference	
Male	1.13 (0.47 – 2.72)	0.781
**Hypertension**		
No	Reference	
Yes	1.41 (0.66 – 3.00)	0.374
**Diabetes mellitus**		
No	Reference	
Yes	0.48 (0.08 – 2.92)	0.429
**Cardiovascular disease**		
No	Reference	
Yes	Not estimable	
**Smoking history**		
No	Reference	
Yes	1.66 (0.78 – 3.56)	0.191
**Alcohol consumption**		
No	Reference	
Yes	1.00 (0.45 – 2.24)	0.991
**Tumor location**		
Lower	Reference	
Middle	1.97 (0.80 – 4.87)	0.141
Upper	1.74 (0.66 – 4.64)	0.265
**Tumor length, cm**		
<3	Reference	
≥3	1.90 (0.73 – 4.91)	0.188
**Surgical approach**		
Ivor Lewis	Reference	
Mckeown	1.87 (0.51 – 6.85)	0.348
Sweet	1.48 (0.39 – 5.67)	0.566
**Clinical T stage**		
1	Reference	
2	0.83 (0.29 – 2.39)	0.731
3	6.64 (0.94 – 46.73)	0.057
**Clinical N stage**		
0	Reference	
1	2.73 (0.98 – 7.67)	0.056
**RLN lymph node retrieval**		
RLN lymph node retrieval ≥ 1	Reference	
RLN lymph node retrieval = 0	3.53 (1.70 – 7.31)	<0.001
**Pathologic T stage**		
pT1b	Reference	
pT2	2.10 (0.86 – 5.10)	0.102
**Receipt of adjuvant therapy**		
No	Reference	
Yes	0.51 (0.23 – 1.15)	0.106
**Anastomotic leakage**		
No	Reference	
Yes	0.76 (0.36 – 1.60)	0.466
**Respiratory complications**		
No	Reference	
Yes	1.02 (0.57 – 1.85)	0.941

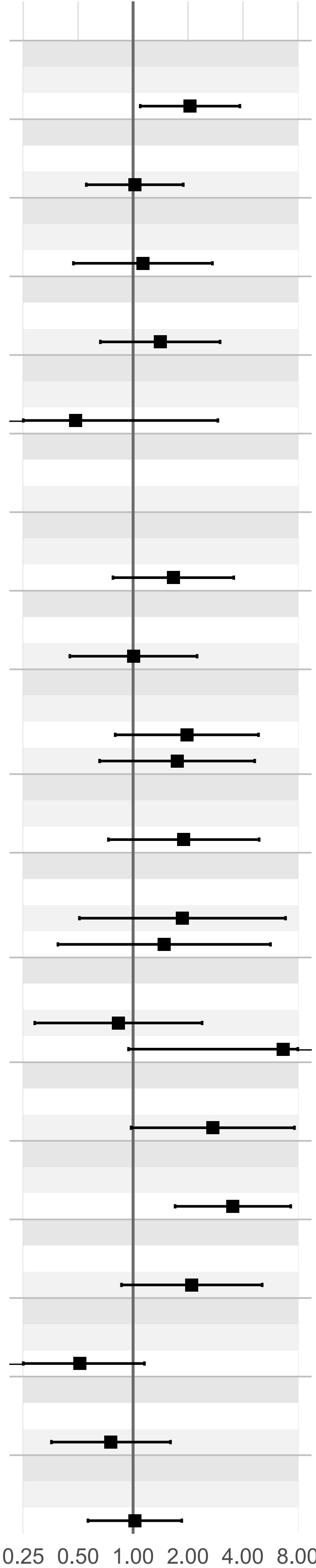

Supplement: Supplemental Information 2 — Forest plot of Cox proportional hazards model for overall survival (OS) in a sensitivity analysis that additionally adjusted for postoperative factors, including recurrent laryngeal nerve palsy, anastomotic leakage, respiratory complications, and receipt of adjuvant therapy. Hazard ratios (HRs) with 95% confidence intervals (CIs) are presented for RLN LN resection status and covariates included in the model. [file peerj-14-21293-s002.pdf]
