# Supplementary material for: Association of recurrent laryngeal nerve lymph node retrieval with survival in early-stage resectable esophageal squamous cell carcinoma: a retrospective cohort study
Source: PeerJ. 2026 Jun 4;14:e21293. doi: 10.7717/peerj.21293 (PMC13242742; doi:10.7717/peerj.21293)

Variables	Adjusted HR (95% CI)	P
**Age**		
<65	Reference	
≥65	1.74 (0.96 – 3.16)	0.066
**Body mass index**		
<24	Reference	
≥24	1.06 (0.58 – 1.95)	0.845
**Sex**		
Female	Reference	
Male	1.19 (0.55 – 2.58)	0.662
**Hypertension**		
No	Reference	
Yes	1.14 (0.57 – 2.30)	0.708
**Diabetes mellitus**		
No	Reference	
Yes	0.48 (0.09 – 2.61)	0.398
**Cardiovascular disease**		
No	Reference	
Yes	Not estimable	
**Smoking history**		
No	Reference	
Yes	1.13 (0.52 – 2.45)	0.758
**Alcohol consumption**		
No	Reference	
Yes	1.51 (0.68 – 3.35)	0.306
**Tumor location**		
Lower	Reference	
Middle	2.56 (1.05 – 6.23)	0.039
Upper	2.36 (0.89 – 6.22)	0.083
**Tumor length, cm**		
<3	Reference	
≥3	1.89 (0.79 – 4.53)	0.154
**Surgical approach**		
Ivor Lewis	Reference	
Mckeown	2.01 (0.64 – 6.28)	0.229
Sweet	1.28 (0.38 – 4.28)	0.692
**Clinical T stage**		
1	Reference	
2	0.79 (0.27 – 2.28)	0.663
3	12.30 (1.88 – 80.60)	0.009
**Clinical N stage**		
0	Reference	
1	3.54 (1.49 – 8.43)	0.004
**RLN lymph node retrieval**		
RLN lymph node retrieval ≥ 1	Reference	
RLN lymph node retrieval = 0	3.95 (1.98 – 7.87)	<0.001
**Pathologic T stage**		
pT1b	Reference	
pT2	2.27 (0.94 – 5.52)	0.070
**Receipt of adjuvant therapy**		
No	Reference	
Yes	0.65 (0.32 – 1.34)	0.246
**Anastomotic leakage**		
No	Reference	
Yes	0.71 (0.35 – 1.42)	0.334
**Respiratory complications**		
No	Reference	
Yes	1.23 (0.69 – 2.20)	0.477

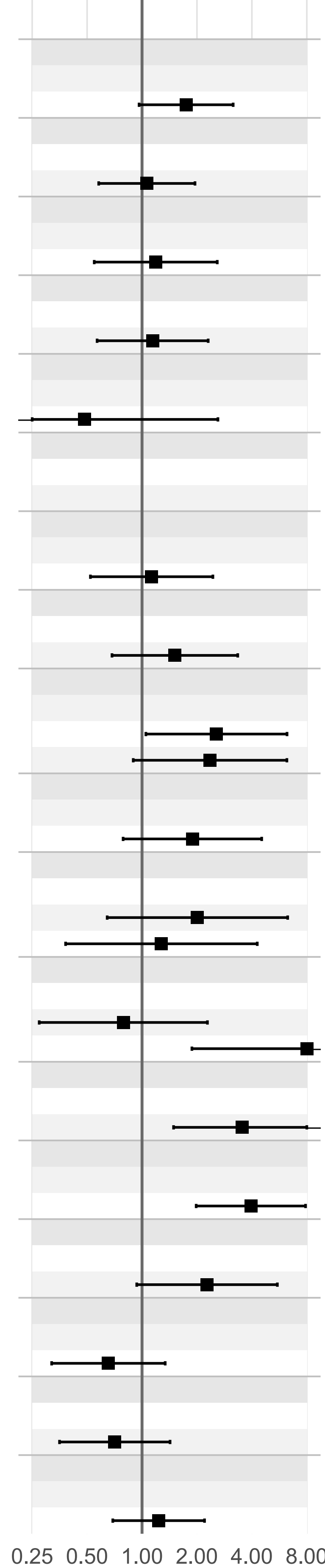

Supplement: Supplemental Information 3 — Forest plot of Cox proportional hazards model for disease-free survival (DFS) in a sensitivity analysis that additionally adjusted for postoperative factors, including recurrent laryngeal nerve palsy, anastomotic leakage, respiratory complications, and receipt of adjuvant therapy. Hazard ratios (HRs) with 95% confidence intervals (CIs) are presented for RLN LN resection status and covariates included in the model. [file peerj-14-21293-s003.pdf]
