# Supplementary material for: Association of recurrent laryngeal nerve lymph node retrieval with survival in early-stage resectable esophageal squamous cell carcinoma: a retrospective cohort study
Source: PeerJ. 2026 Jun 4;14:e21293. doi: 10.7717/peerj.21293 (PMC13242742; doi:10.7717/peerj.21293)
Supplement: Supplemental Information 5 — Unweighted Kaplan–Meier curves for procedure-related subgroup analyses. Panels A–B compare unilateral versus bilateral RLN LN retrieval; panels C–D compare RLN LN sampling versus formal RLN LN dissection. HRs and P values are from unadjusted Cox models. [file peerj-14-21293-s005.pdf]

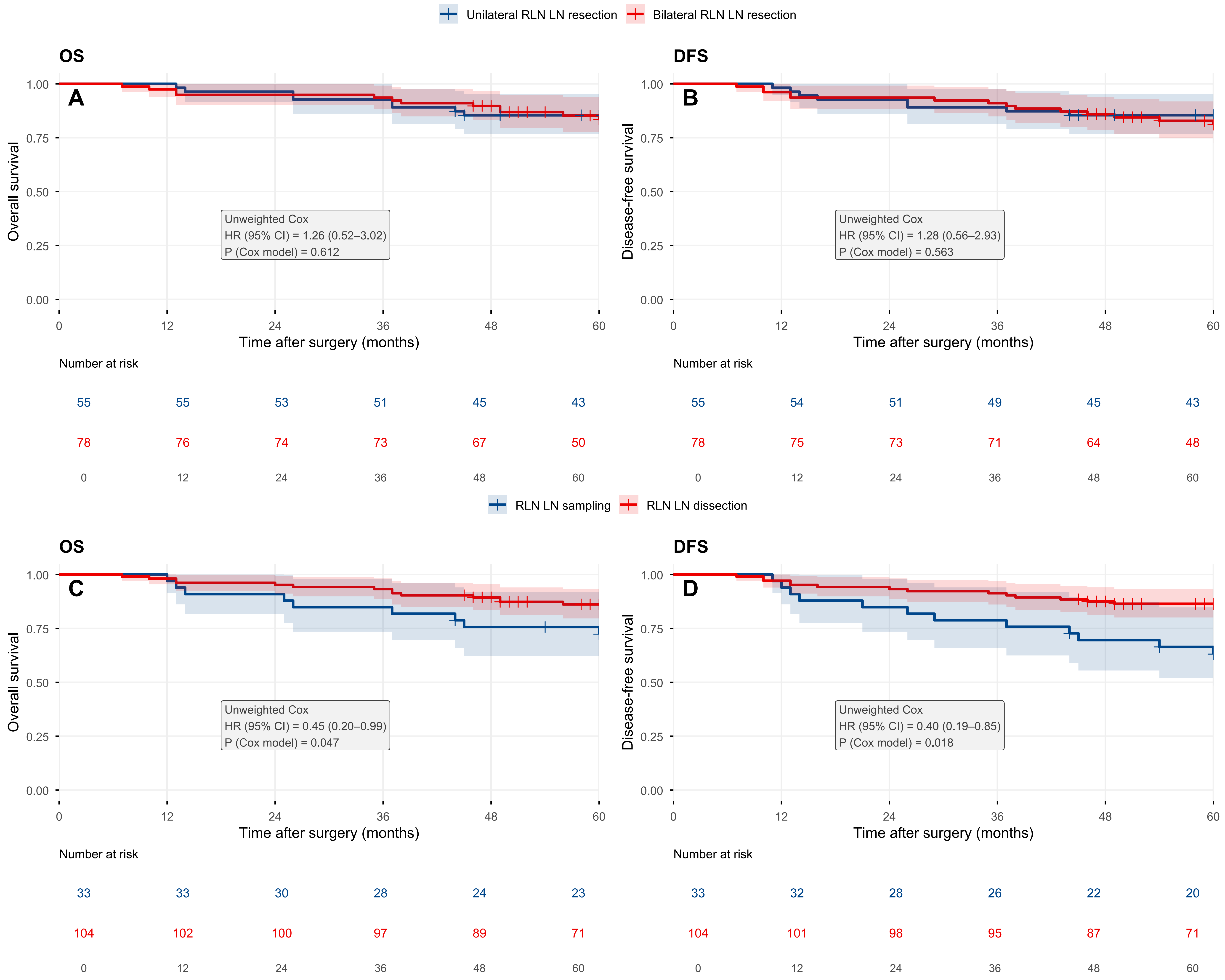

RLN LN: recurrent laryngeal nerve lymph node.
